# High mortality among male HIV-infected patients after prison release: ART is not enough after incarceration with HIV

**DOI:** 10.1371/journal.pone.0175740

**Published:** 2017-04-28

**Authors:** Florence Huber, Alice Merceron, Yoann Madec, Gueda Gadio, Vincent About, Agathe Pastre, Isabelle Coupez, Antoine Adenis, Leila Adriouch, Mathieu Nacher

**Affiliations:** 1 COREVIH, Cayenne General Hospital, Cayenne, French Guiana, France; 2 Day Hospital, Cayenne General Hospital, Cayenne, French Guiana, France; 3 Reseau Kikiwi, Cayenne, French Guiana, France; 4 Faculty of Medecine Hyacinthe Basturaud, University of French Guiana, Cayenne, French Guiana, France; 5 Emerging Diseases Epidemiology Unit, Pasteur Institute, Paris, France; 6 Centre d’Investigation Clinique Epidemiologie Clinique Antilles Guyane, INSERM CIC 1424, Cayenne General Hospital, Cayenne, French Guiana, France; 7 UCSA, Cayenne General Hospital, Cayenne, French Guiana, France; Medizinische Universitat Wien, AUSTRIA

## Abstract

**Context:**

French Guiana is a South American French territory, where HIV prevalence consistently exceeds 1% in the adult population. In the only correctional facility, HIV prevalence fluctuates at around 4%.

**Aims:**

After describing the population of HIV-positive inmates, we aimed to evaluate mortality after release from the correctional facility, and to identify its predictive factors.

**Rationale:**

Outside North American settings, data on treatment outcome and vital status of HIV-positive former inmates are scarce. There were no data in French Guiana. Filling this gap represents a basis for potential improvements.

**Methods:**

All HIV-infected adults released from an incarceration of 30 days or more, between 2007 and 2013, were enrolled in a retrospective cohort study. Mortality was described over time, one to seven years following release, using Kaplan-Meier estimates. Factors associated with mortality were identified through a non-parametric survival regression model.

**Results:**

147 former inmates were included. The male to female ratio was 4.4. The median age was 37.3 years. The majority were migrants, 25.8% were homeless, 70.1% suffered from substance abuse, with 34.0% of crack-cocaine users. On admission, 78.1% had an early HIV-stage infection (CDC-stage A), with a median CD4 count of 397.5/mm3, 34.0% had one comorbidity, mainly hypertension. Upon release, 50.3% were on ART. Reasons for not being treated were not fulfilling the criteria for 74.6%, and refusing for 15.1%. Before release, 84.5% of the patients on ART had a viral load≤200cp/ml. After release, 8.2% of the cohort had died, with a crude incidence of 33.8/1000 person-years. All recorded deaths were males, with an incidence of 42.2/1000 person-years. Comparing with the age-specific mortality rates for males in French Guiana, the standardized mortality ratio was 14.8. In multivariate analysis, factors associated with death were age and CD4 count before release.

**Conclusion:**

Despite access to ART while incarcerated, with good virological outcome, the post-release mortality was very high for males, almost 15 times what is observed in the general male population living in French Guiana, after age standardization. Access to ART in correctional facilities may be a necessary, but not sufficient condition to protect male inmates from death after release.

## Introduction

French Guiana is a French overseas territory located in South America, known to have the highest HIV prevalence among French territories, exceeding 1% among pregnant women for over 20 years [1]. Among the French-Guianese population, some sub-populations have been identified to be at higher risk of acquiring HIV: crack drug-users, commercial sex-workers, immigrants, and low educational level people. In France, as elsewhere, inmates often accumulate these vulnerabilities [1,2].

Inmates are known to have a much higher HIV-prevalence than the general population, reaching up to 50 times the general population prevalence [3,4]. In French Guiana, HIV prevalence among inmates was 3.9% in 2014 [5], which is around 4 times higher than the general population, and twice the prevalence reported in the French national correctional facility survey [6].

Incarceration may have a positive effect on the course of HIV infection, giving an opportunity to diagnose unknown HIV infections and to start anti-retroviral therapy (ART) [7]. Virological success can be achieved in a correctional facility (CF), promoted by a structured environment and steady clinical follow-up [8]. Nevertheless, the end of incarceration is often associated with poor retention in care and treatment interruption, losing the health benefits of the previous incarceration, with an increase of the viral load and loss of CD4 cells between two incarceration periods [9–12].

Outside North American settings, data on treatment outcome and vital status in the years following release are scarce. The purpose of our study was thus to evaluate the outcome of people living with HIV (PLWHIV) after release from the French Guiana correctional facility, through a retrospective cohort.

## Materials and methods

### Setting and study population

French Guiana is a French territory, located in South America, surrounded by Surinam and Brazil, at 7100 kilometers from the French mainland. The official population is estimated at around 240 000 inhabitants [13]. The socio-economic indicators are particularly bad for a French territory, with an unemployment rate of 22.3%, which is much higher than in the French mainland [14]. Nevertheless, as a French department, the regional gross domestic product is higher than the surrounding countries, and French Guiana remains a land of migration, mainly for economic purposes. Foreign migrants, mostly originating from the Caribbean and South America, represent more than 40% of the adult population [14,15]. Among the PLWHIV followed in the main French Guianese hospital en 2011, 76.6% were foreigners [16].

In French Guiana, the standard of care for PLWHIV is the same as in any other French territory: medical follow-up with specialized infectious disease physicians, routine viral load and CD4 monitoring every 3 to 6 month, depending on the immune-suppression level, free medical care, drugs (including for opportunistic infections and prophylaxis), and laboratory monitoring for all patients. Thus, anti-retroviral drugs are available after medical prescription for illegal immigrants and prisoners. Since September 2013, anti-retroviral drugs are offered to any PLWHIV, according to the French guidelines [17]. Before this date, at the time of the study, criteria for ART initiation were CD4 less than 500 per mm3 if asymptomatic [18].

Medical follow-up is offered by specialists, mainly through the infectious diseases and internal medicine units of the 3 public hospitals (Centre Hospitalier de Cayenne, Centre hospitalier de l’Ouest Guyanais and Centre Medico-chirurgical de la Croix-Rouge de Kourou). Emergency care and hospitalization are offered free-of-charge to patients in need, in these 3 hospitals.

The Remire-Montjoly CF is the only place of incarceration in French Guiana. It includes a prison for males (“centre de détention”, for inmates sentenced for longer than two years), a jail for males and a jail for women (“maison d’arrêt”, for inmates awaiting trial or sentenced for a duration shorter than two years). In January 2014, its capacity was 614 places for 729 inmates incarcerated. At the first medical check-up, HIV and hepatitis B and C testing are offered as a provider-initiated testing and counselling strategy, using an opt-out policy [19,20]. Refusal is rare. Around 30 PLWHIV are usually followed on a routine base. A medical team offers clinical follow-up and outpatient care to all inmates at the CF medical centre. After referral, an infectious disease specialist offers consultations for HIV care, at the same site. At the time of release, HIV-positive inmates receive a provision of drugs (one week treatment was given before 2014), and they are referred to the specialized units. The prescribed treatment is delivered free of charge.

### Study design and data sources

A retrospective cohort was constituted, enrolling all HIV-infected adults (≥18 years) released from the Remire-Montjoly CF between January 2007 and December 2013, after incarcerations of more than 30 days. In case of multiple incarcerations during this period, the last one was considered as the index incarceration. The data collection was performed at the end of 2014. Thus, all included patients had at least an observational period of a year, up to a maximum follow-up of seven years.

The main source of data was the electronic medical records (EMR) NADIS, used for HIV follow-up at the regional French Guianese level. Patients were asked to sign a consent form before their data were included in NADIS. All registered infectiologists in French Guiana had access to the regional EMR for the post-release follow-up. Additional data were collected from paper medical files used for routine follow-up by the three general practitioners in charge of the detainee’s medical care, and the registries of the three regional hospitals. The mortality registries of the main towns of French Guiana (covering 80% of the French Guianese population) were also consulted.

These sources were used to collect the data regarding:

- Socio-demographic characteristics (including place of birth, and type of housing prior to incarceration)
- Past incarcerations, date of entry and release of the index incarceration
- Medical and psychiatric background, substance abuse, HIV history, comorbidity
- HIV-clinical stage, opportunistic infections, immunological and virological results at the beginning and the end of the index incarceration
- Post-release events, including linkage to specialized care, hospitalisations, outpatient care in the emergency departments, occurrences of opportunistic infections (IO) or any CDC-stage C events, further incarcerations
- Date of death and other pejorative events when applicable.

All data were recorded manually, through an anonymous identification format, by a medical doctor, with respect of confidentiality. The study protocol was reviewed and approved by the Ethics Committee of Cayenne Hospital. The data base was declared to the Commission Nationale de l’Informatique et des Libertés (CNIL), under the number 1975083v0.

### Outcomes

The primary outcome was to evaluate the incidence of mortality among former HIV-positive inmates released from French Guiana CF between 2007 and 2013. The secondary outcome was to describe the characteristics of PLWHIV incarcerated in French Guiana CF between 2007 and 2013, and to identify the factors associated with the death of former HIV-positive inmates.

### Statistical analyses

Mortality after release was described, using Kaplan-Meier estimates. The analysis to identify factors associated with mortality was restricted to men, as no death was recorded among women, who moreover represented a small proportion of the sample. A parametric regression model, with an accelerated failure time approach was used, as the proportional risk hypothesis was not fulfilled for several variables.

Some patients had no identified contact with the health system after release. We used a logistic regression to better understand the profile of this subpopulation.

For both multivariate analyses, factors associated with the outcome were entered in the model if p-value was less than 0.25 in univariate analysis. A stepwise backward procedure was then performed to identify factors independently associated with the outcome.

Statistical analyses were performed with Stata 11 (Stata Corporation, college station, Texas, USA).

## Results

### Study population

Among 160 PLWHA released from incarceration during the study period, 147 HIV-infected adults met the inclusion criteria and were included in the cohort. A vast majority were migrants and males. Country of birth was one of the neighboring countries: Guyana, Surinam and Brazil for 64.0% of the cases. The male to female ratio was 4.4. The median (inter quartile range (IQR) age was 37.3 (31.0–43.6) years. The socio-economic status was low and a vast majority suffered from substance abuse (Table 1). A majority of patients, 64.6%, had previously been incarcerated. The median (IQR) number of previous incarcerations was 1.0 (0–4). The median (IQR) duration of the index incarceration was 9.0 (4.3–18.2) months.

Opioids consumption was rare, but 34.0% of the inmates declared using crack-cocaine. These patients were often born in France (p<0.001), homeless (p<0.001), and had a higher mean of previous incarceration than their counterparts: 4.8 (95%CI: 3.4–6.1) versus 1.4 (95%CI: 1.0–1.8).

**Table 1:** Characteristics of the study population (N = 147).

		n	%
**Sex**	Males	120	81.6
	Females	27	18.4
** Age category (years)**	Between 18 and 30	30	20.4
	Between 30 and 40	63	42.9
	More than 40	54	36.7
**Country of birth**	Guyana	58	39.5
	France (including French Guiana)	46	31.3
	Surinam	22	15.0
	Brazil	14	9.5
	Other	7	4.7
Qualification level*	Never went to school	67	57.3
	Primary school	17	14.5
	Junior secondary school	25	21.4
	High-school	8	6.8
**Type of housing**	Homeless	38	25.8
	Gave an address	92	69.6
	Not provided	17	11.6
**Time of diagnosis of HIV**			
	Before the index incarceration		
	While incarcerated	55	37.4
	Outside prison/jail	64	43.6
	During the index incarceration	28	19.0
**Psychiatric disorders**		**38**	**25.8**
	Psychosis	13	8.8
	Depression	13	8.8
	Anxiety	16	10.9
	Suicide attempt	4	2.7
**Substance abuse**		**103**	**70.1**
	Tobacco	77	52.4
	Marijuana	66	46.9
	Crack	50	34.0
	Alcohol	38	25.9
	Heroin	3	2.0
**Other comorbidity**		**50**	**34.0**
	Hypertension	17	10.9
	Overweight	10	6.1
	Diabetes	7	4.8
	Chronic renal failure	4	2.7
	Epilepsy	4	2.7
	Asthma	5	3.4
	Others	7	4.9
**Viral co-infection**	Positive HBs Antigen	12	8.2
	Positive anti-CHV Anti-bodies	6	4.1
	Positive HTLV1 Anti-bodies	7	4.8
**ART when incarcerated**			
	Naive	86	58.5
	Interrupted	17	11.6
	Ongoing	44	29.9

*24 missing observations (17.0%), denominator: n = 117.

Most inmates (56.5%) were initially tested positive during incarceration. This was particularly true for migrants (62.4%), compared to individuals born in France (43.5%, p = 0.03). The HIV- positive status was known since a mean of 5.7 years (IQR: 2.3–8.7, median: 5.2). This duration was the longest for patients born in France (7.8 years, 95%CI: 6.2–9.3), and the shortest for those born in Surinam (4.0 years, 95%CI: 2.5–5.6). Before index incarceration, 17.0% of patients had a previous opportunistic infection, mostly tuberculosis (10.9%) and disseminated histoplasmosis (4.1%). A vast majority of inmates had an early HIV-stage infection: 78.1% were CDC-stage A (asymptomatic, or acute HIV, or persistent generalized lymphadenopathy), 4.8% stage B (symptomatic conditions, not stage A or AIDS), and 17.1% stage C (AIDS-indicator conditions). The median (IQR) CD4 count on admission was 397 (273–565) cells/mm3. Around one third had one comorbidity, mainly hypertension.

At the time of incarceration, 29.9% of the inmates were taking an ARV treatment, whereas, at the time of release, 50.3% were taking an ARV treatment and received a prescription for the outside (n = 74). For the untreated patients, the reasons were the absence of treatment indication for 74.6%, and refusal to start ART while incarcerated for 15.1%.

Considering the whole cohort, the mean CD4 count did not differ between the first and last measurement (p = 0.52). Nevertheless, a clear virological response was observed: 24.7% had a viral load ≤200 copies/ml on admission, versus 45.9% when discharged (p<0.001). Restricting to patients on ART with available data (n = 71), 84.5% had a VL≤200 copies/ml, and their mean CD4 increase was 53.8/mm3 (-16.9–124.5). When the index incarceration was longer than a year, 93.7% of patients on ART had a VL≤200 copies/ml.

During the index incarceration, 18.5% patients had been hospitalized (n = 27). This was linked to HIV for one third of them. A new AIDS-defining event was diagnosed in 9 cases (6% of the cohort), mostly tuberculosis.

### Outcomes after release

**Fig 1:**
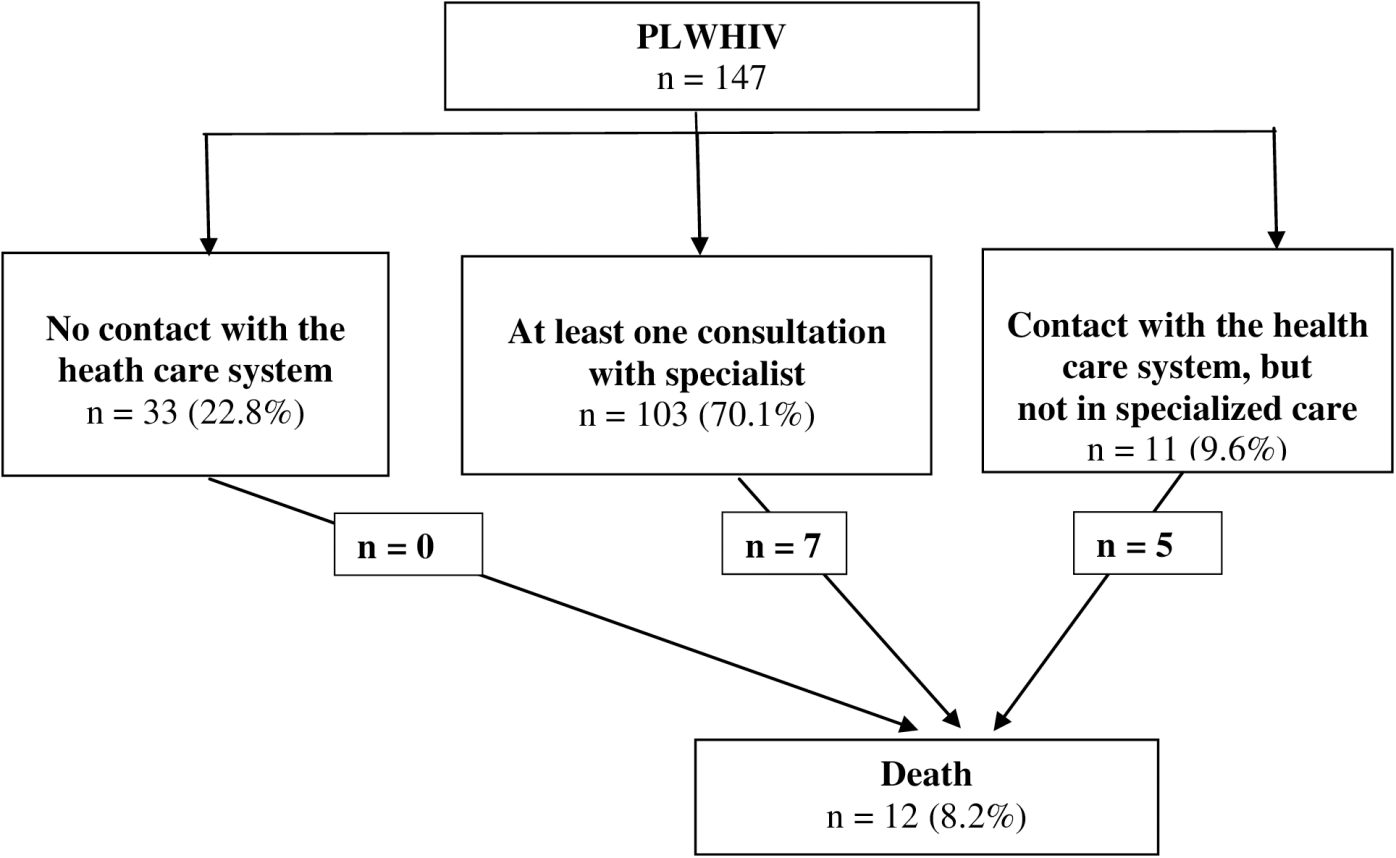
Patient flow, within the 7 years after release from the French Guiana CF.

After tracing, no data were found for 22.8% of the cohort (Fig 1). Multivariate analysis showed that these patients were often migrants (aOR = 4.6, p = 0.02), not on ART when released (aOR = 22.6, p<0.001), did not report being a crack user (aOR = 6.0, p = 0.006), and they had early stage HIV-disease, defined by CDC-stage A with a NADIR of CD4>200/mm3 (aOR = 5.1, p = 0.025).

Overall, 12 patients died, 6 deaths could be linked to the evolution of HIV-disease, another one was a homicide, one a stroke. We had no information on cause of death for 4 patients. All deceased patients were males and 8 among 12 were taking ART on release. Nevertheless, only 2 of the 12 patients who died were taking ART and controlling their viral load before they died (one died from a stroke, one from a T-cell lymphoma). For the 10 other deaths: 6 patients had interrupted ART or were known for severe adherence issues, 4 patients were treatment naive.

Mean duration between release and death was 28.1 months. One year after release, 3.7% (1.4–9.6) of the patients were found dead, 10.5% (10.5–19.5) after 3 years, 15.6% (8.5–27.8) after 5 years, 23.3% (11.0–45.2) after 7 years.

Death incidence rate was estimated at 33.8 (19.2–59.6) per 1000 person-years. Restricting analysis to males, this was 42.2 (24.0–74.3) per 1000 person-years. Comparing with the age-specific mortality rates of the French Guianese male population in 2013 [21], the standardized mortality ratio was 14.8 for HIV-positive former inmates.

Univariate analysis showed that the CD4 count levels before release were lower for those who died (p = 0.01): mean 285.4/mm3 (median: 251.5), versus 459.3/mm3 (median: 408.0). Age and time since HIV diagnosis did not differ between those found dead and others (respectively p = 0.99 and p = 0.88).

Due to the absence of deaths among females, we restricted the multivariate analysis to males. Overall, there were 284.5 person-years of observation. Results are shown in Table 2.

**Table 2:** Risk-factor for death following release from incarceration, among men who left French Guiana CF between 2007 and 2013 (parametric regression model, with an accelerated failure time approach, n = 120).

	Death	Crude Time ratio	p	Adjusted Time-Ratio	p
	n (%)	(95%CI)			
**Age > 37 years old**					
Yes	10 (15.4)	0.12 (0.01–1.08)	0.06	0.09 (0.01–0.9)	0.04
No	2 (3.6)	Reference		Reference	
**Last CD4 result before release**					
< 200/mm3	6 (30.0)	0.08 (0.01–0.61)	0.01	0.07 (0.01–0.50)	< 0.01
≥ 200/mm3	6 (6.6)	Reference		Reference	
Advanced HIV-disease*					
Yes	8 (17.4)	0.16 (0.02–1.01)	0.05	—	
No	4 (5.4)	Reference			
**Declared an address**					
Yes	4 (5.5)	4.90 (0.63–37.89)	0.12	4.24 (0.66–27.31)	0.13
No	8 (17.0)	Reference		Reference	
**Born in a French territory**					
Yes	7 (17.1)	0.31 (0.05–2.02)	0.22	—	
No	5 (6.3)	Reference			
**Previous incarceration**					
Yes	7 (8.7)	4.07 (0.67–24.52)	0.13	—	
No	5 (12.5)	Reference			

* CDC-stage C and/or NADIR of CD4 <200.

Univariate analyses were also performed for: HIV-diagnosed in detention, previous incarceration, ARV treatment on release (ongoing, newly initiated, none), substance abuse, known psychiatric disorders, having kids, duration of the index incarceration. None of the relations were significantly significant. Qualification level was not included as 17% of this data was missing, and its reliability was questioned (definition not standardized between countries).

## Discussion

As described in the United States, HIV-prevalence in the French Guiana CF was higher than in the general population [21], four times greater than the regional prevalence.

The population of PLWHIV included in our survey had characteristics already described in North-American surveys: around 80% of men, often aged between 30 and 50 years old, with a low educational background, often belonging to minority communities, and characterized by a high prevalence of psychiatric disorders and substance abuse [8,22–25]. When compared with the general population of inmates incarcerated in French Guiana, our studied population seemed to have more female (18.4% versus 10%), to be older, and include a higher proportion of drug-users [26]. These characteristics were already described in the Connecticut correctional facilities, where HIV-positive prisoners were more likely to be female, older, to belong to a racial or ethnic minority, and to be a substance abuser [11].

As reported elsewhere, opt-out HIV screening seemed an efficient strategy to diagnose HIV among inmates [7,27,28]. It allowed diagnosing a high number of early stage infections, with no physical signs of immune depression (nearly 80% were CDC-stage A).

In our survey, most of the inmates had been diagnosed while incarcerated (56.5%). This rate seemed relatively high, compared with the 24% reported in the French national survey PREVACAR [6]. Our result may be linked to the high proportion of migrants in our cohort (68.7%). With a more difficult access to prevention and care, migrants may benefit even more from HIV screening in the correctional facility: almost 2/3 were diagnosed during imprisonment, which was higher than for French natives. Moreover, very high prevalence had been reported among some migrant population groups in French correctional facilities (e.g.:15.4% [6.6–31.8] for inmates born in sub-saharian Africa) [6]. This also seems the case in French Guiana, as the prevalence among inmates born in Guyana is estimated around 10%.

Despite the good virological response while incarcerated for those on ART, a high proportion of former inmates died in the few years following release. It has been described elsewhere that the medical benefit of incarceration was lost after release from the correctional facility, with a viral rebound and loss of CD4 [7,9,11,12]. To our knowledge, the incidence of AIDS-specific mortality has been rarely reported among HIV-positive former inmates. Indeed, after searching the pubmed database, using keywords “(mortality OR death) AND (HIV* OR AIDS) AND (release* OR "former inmate*" OR "post-release") AND (jail* OR prison* OR "correctional facilit*")”, we found 40 references but no report of death incidence among HIV-positive former inmates.

Despite the wide access to treatment and care, following the national French guidelines, and despite the low proportion of PLWHIV with advanced HIV-disease, the mortality rate was extremely high among males in our cohort. Indeed, the standardized mortality ratio was 14.8, compared with the male mortality rate in French Guiana, 2013. Moreover, it seemed that ART prescription on release was not enough to protect against death, as most of the patient who died were on ART when released (8/12), but not able to maintain the treatment (6/8). The observed male mortality rate was very close to the overall mortality rate reported before highly active ART was available in 1996. Since then, in industrialized countries,the risk approached that in the general population [29].

In the medical literature, non-infectious causes of death were over-represented among former inmates, whether HIV-positive or not, in particular drug-related deaths, homicides, suicides, accidents, cardio-vascular events, lung or liver cancer, and viral hepatitis [30–32].

Of 30 237 former inmates released from the Washington State prison, the mortality rate was 7.77 deaths per 1000 person-year [33]. Our result was 4.4 times higher than this, and around 10 times higher than the rate of the general French Guianese population. Some striking comparisons emphasize the importance of the death rate in our cohort: among marines in Irak in 2003–2006, the mortality rate was 9 times lower (3.92 per 1000 person-years), and our male mortality rate was twice that of the American troops during the Vietnam War [34].

HIV-positive former inmates may combine the risk of death of former inmates, to which are added the risk of AIDS-defining events. Indeed, a high proportion of our cohort had one or more cardiovascular risks (10.9% suffered from hypertension, 52.4% were smokers), and only half of the deaths could be directly linked to HIV.

**Fig 2:**
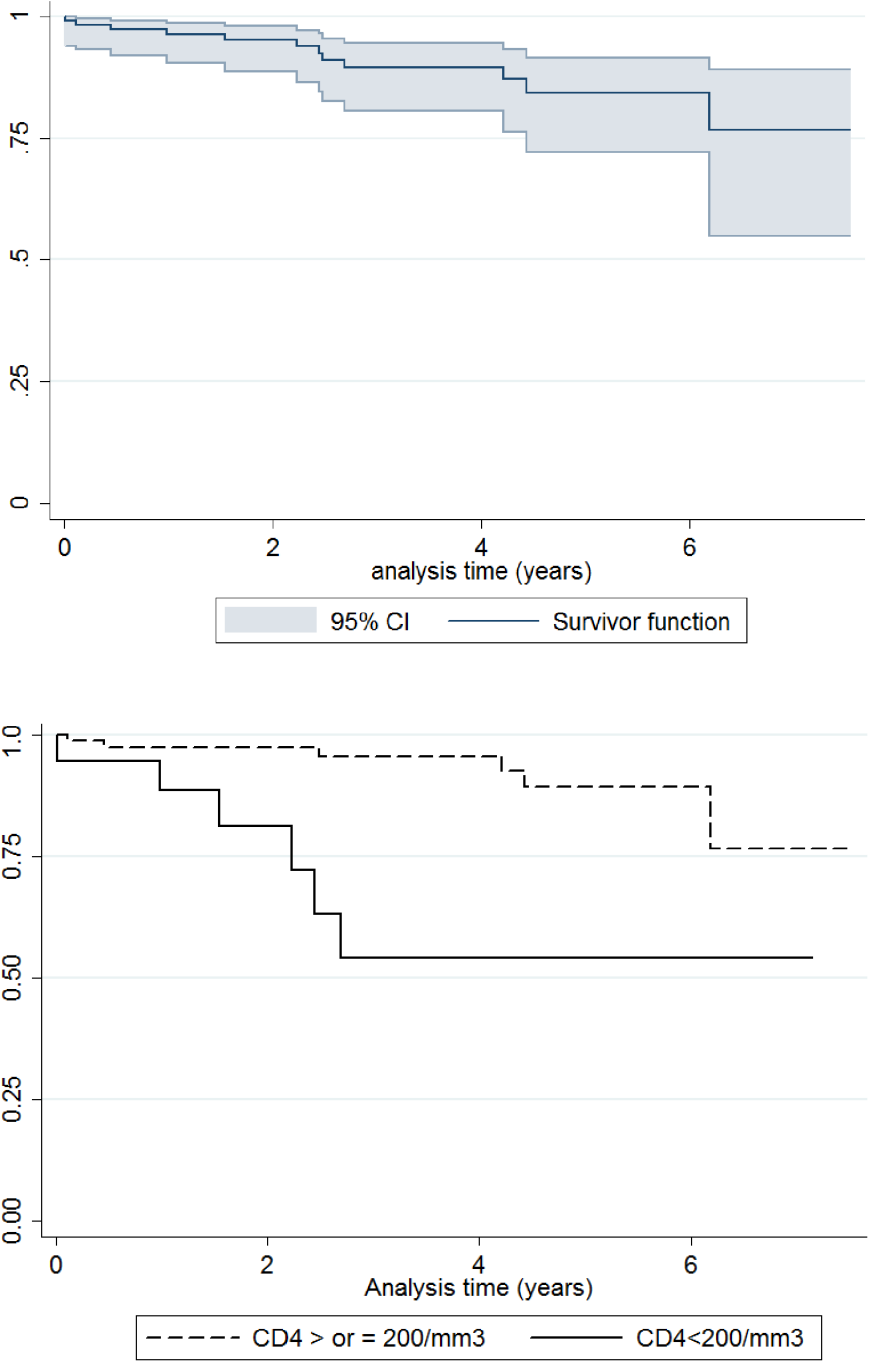
Mortality following release from the French Guiana CF (Kaplan-Meier survival estimate), overall, and with CD4 at less than 200/mm3.

In our cohort, the last CD4 count before release was the strongest factor associated with death in multivariate analyses (Table 2). Almost 50% of the patients with less than 200 CD4/mm3 died, and in all cases, these deaths occurred quickly, within the 3 years following release (Fig 2). Being older than 37 years was also independently associated with death in multivariate analyses. This finding is still consistent with Baillargeon surveys among Texas HIV inmates, published 17 years ago [35,36].

The role of gender seems less clear, and may require further investigation. In our cohort, males may be more vulnerable than females, as no death was found among women. In the studies mentioned above, Baillargeon stated than women had a survival advantage compared to men. This was consistent with another survey performed in the Connecticut prison system, where women had better virological outcomes than men [11]. Nevertheless, being a male was associated with a two-fold higher likelihood of being retained in care after release in another study [24]. Independently from correctional environment, data are also contradictory [26,34,37].

In our survey, the proportion of individuals who reported to be homeless before being incarcerated was around 25%. The rate was probably higher than this, because 30% did not report any address. A prevalence of homelessness as high as 42% has been reported in the United States [38]. Former homeless persons were more often cocaine and multiple drug consumers than other inmates (p<0.001), with a higher prevalence of alcohol use and severe psychiatric illnesses [38]. As described by Nadine Chen, former inmates with triple diagnosis -substance abuse, psychiatric disorder, homelessness- are often engaged in a “revolving cycle between homelessness and criminal justice”. In our setting, the triple burden homeless, multi-incarceration, cocaine-use was well represented. Cocaine, mainly smoked as crack, is known to be associated with poor outcomes in French Guiana, with an adjusted hazard ratio of 3.8 (1.9–7.5) for developing AIDS, when compared with non-users [39]. Nevertheless, substance abuse was not correlated with mortality, whether in uni- or multivariate analysis. Previous incarcerations and having declared an address had a protective effect in univariate analysis, but this effect was no longer significant in multivariate analysis. From a health perspective, it seems in theory possible that multiple incarcerations would have a protective effect for the triple burden subpopulation “homeless-addicted-multi-incarcerated”, through access to care and re-initiation of ART while incarcerated. Nevertheless, our small sample size may limit the statistical power to show that.

Concerning housing status, the adjusted time-ratio was strongly protective from death, for those who declared an address, but this result was no longer significant in multivariate analysis (p = 0,12). Here again, this may be due to lack of statistical power, but maybe also to the quality of the retrospective data, regarding non-medical information extracted from medical files, which may have been less reliable than strictly medical data.

The medical literature showed strong evidence that housing status was associated with pejorative outcomes for HIV-positive individuals, including death, poor engagement in care, low adherence to ART and high rate of risk behaviors for viral transmission. Among 152 studies compiled by Aidala et al, 144 showed that housing status was associated with poorer medical care or health outcomes, and 5 studies found that homelessness was associated with an increased risk of premature mortality for PLWHIV [40]. Lieb reported an almost 10-fold increase in the mortality risk (HR = 9.98; 95% CI: 2.34, 42.5) for patients who reported homelessness in the 12 past months, compared to the others [41]. Two randomized controlled trials point in the same direction: in one survey, patients who benefited from housing assistance improved their mental health status (perceived stress, depression) and had a lower use of the emergency department; in both surveys, patients improved their virological outcomes [42,43].

Our study highlighted that ART at the time of discharge is not enough to protect from death, as treatment interruptions seem frequent, and may quickly lead to the death of the most immunodeficient patients (Fig 2). Former inmates face competing priorities which impact adherence and medical follow-up. Inadequate housing, as another component of the social instability, should be tackled, through pre-release discharge planning [7,9,24,38,44]. Pre and post-release interventions have been successfully experienced in the US [25,45,46]. In our setting, these are weak or do not exist. Moreover, one main obstacle faced by HIV-positive migrants is to legalize their administrative status on the French territory, by acquiring a residence permit for medical reasons. This complex administrative process is the first step to enable migrant former inmates to find legal work and housing. Without this condition, no rehabilitation project is feasible in the territory. No social worker was assigned to this task in the only French Guianese correctional facility.

This research, including a time-consuming tracing, could be carried out in the specific territory of French Guiana, and may not have been easily feasible elsewhere. Its results may not be generalized to any other territory. Since 2013, the French ART recommendation has changed toward universal treatment, independently of CD4 counts. It would certainly be of interest to compare the same mortality indicators, as well as retention in care, for patients released before and after 2013.

One main limitation of this study was the lack of statistical power, due to the small size of our sample. Indeed, the trends of our results highlight the possible correlation between housing status and mortality. We aim to investigate the post-carceral social and medical services use in the future, to gain some insights on what has happened since release.

The quality of some data may be questioned, due to the retrospective nature of the study. Data collected from daily clinical reports were not standardized and may not always be reliable, notably for non medical information. Our result may also be subject to selection bias, as we found no information for 22.8% of the patients after their release. They were mostly migrants with early stage infection and not on ART. Some of them possibly went back to their country and may have continued to have a medical follow-up abroad. On the other hand, some deaths were certainly missed; in particular if they occurred in remote areas where we were unable to investigate (we consulted the death registries which covered around 80% of the local population). Moreover, in our vast Amazonian territory, death by homicide can easily be concealed, and thus, would not be registered among our cohort of patients. It is thus difficult to say if the observed mortality rate was over or under-estimated. Nevertheless, its magnitude was strikingly high.

## Conclusion

Our comprehensive study highlights the vital prognosis of HIV-positive inmates released from French Guiana CF before 2013, when ARV treatment was offered to patients with CD4<500/mm3. Incarceration appeared as an opportunity to diagnose HIV infection at an early stage, and to start ART. Migrants and marginalized persons may particularly benefit from HIV-testing in correctional facilities.

Nevertheless, post-release mortality rate was extraordinary high among men, with a standardized mortality ratio at 14.8, compared with the general French Guianese population. As elsewhere, CD4 count and age were associated with the risk of death. Other variables, like housing status, may have a strong impact, but our small sample was not able to show it.

Our study suggests that medical care with ART is not sufficient to help HIV-positive former inmates to stay alive in the years following their release from correctional facilities. As long as resources are not dedicated to the preparation of release and post-release interventions, including the management of administrative matters, our treatment and clinical work may only have a limited impact. The problem goes well beyond the management of HIV infection in the correctional facility, and HIV, definitely, makes it more challenging.
